# Case report: Aortoesophageal fistula—an extremely rare but life-threatening cardiovascular cause of hematemesis

**DOI:** 10.3389/fcvm.2023.1123305

**Published:** 2023-04-20

**Authors:** Alexis Ching Wong, Yu-Mou Chou, Zhong Ning Leonard Goh, Kuang-Fu Chang, Chen-June Seak

**Affiliations:** ^1^ Department of Emergency Medicine, New Taipei Municipal Tucheng Hospital, New Taipei City, Taiwan; ^2^ Department of Emergency Medicine, Lin-Kou Medical Center, Chang Gung Memorial Hospital, Taoyuan, Taiwan; ^3^ College of Medicine, Chang Gung University, Taoyuan, Taiwan; ^4^ Department of Medical Imaging and Intervention, New Taipei Municipal Tucheng Hospital, New Taipei City, Taiwan

**Keywords:** aortoesophageal fistula, haematemesis, upper gastrointestinal bleeding, Chiari's triad, emergency department, computed tomography angiography

## Abstract

Aortoesophageal fistula (AEF) is an extremely rare cardiovascular etiology of hematemesis and upper gastrointestinal bleeding. As such, its recognition and diagnosis are challenging and may be delayed when such patients present to the emergency department (ED). Without timely surgical intervention, AEF is almost always fatal. Awareness of AEF as a possible diagnosis and consequently early identification of these patients presenting to the ED are therefore crucial in optimizing clinical outcomes. We report a 45-year-old male presenting to the ED with the classical triad of an AEF (Chiari's triad)—midthoracic pain or dysphagia, a sentinel episode of minor hematemesis, then massive hematemesis with risk of exsanguination. The case report highlights the importance of considering the differential diagnosis of AEF when evaluating patients presenting to the ED with hematemesis, especially if they have predisposing risk factors such as prior aortic or esophageal surgeries, aortic aneurysms, or thoracic malignancies. Patients suspected of having AEF should be prioritized for early computed tomography angiography to expedite diagnosis and treatment.

## Introduction

Gastrointestinal bleeding is a common presentation seen in the emergency department (ED). Upper gastrointestinal bleeding (UGIB), in which the source of bleeding is proximal to the ligament of Treitz, accounts for approximately 70%–80% of all gastrointestinal hemorrhages (1). UGIB typically manifests as hematemesis, occasionally accompanied by hematochezia and melena. Since peptic ulcer disease (i.e., non-variceal) and esophageal varices represent the vast majority of UGIB etiologies, the possibility of vascular abnormalities is often overlooked (2).

One such rare vascular etiology causing hematemesis is aortoesophageal fistula (AEF). AEFs can be classified as primary or secondary. Primary AEFs directly originate from the native aorta due to various circumstances such as aortic aneurysm (54.2%), foreign body ingestion (19.2%), and advanced esophageal carcinoma (17.0%), in addition to radiotherapy and infections (e.g., syphilis, tuberculosis); secondary AEFs are sequelae of prior vascular interventions such as thoracic aortic or esophageal surgeries (4.7%) and graft placement (3, 4). We report a patient with underlying esophageal cancer who presented to the ED with hematemesis and was subsequently diagnosed with a primary AEF.

## Case report

A 45-year-old Chinese male with underlying recently diagnosed squamous cell carcinoma of the esophagus (stage T4bN2M0) presented to the ED with frank hematemesis. He was hypotensive (blood pressure 95/51 mmHg) and tachycardic (pulse rate 129 beats/min) on ED arrival, while point-of-care full blood count revealed gross anemia (Hb 3.6 g/dl). The patient was resuscitated accordingly with intravenous fluid boluses pending activation of a massive transfusion protocol. He was also treated for the provisional diagnosis of UGIB secondary to bleeding esophageal tumor with tranexamic acid and proton pump inhibitors. The other hematological and biochemical blood investigations returned normal. Further review of his past medical records revealed that the patient had just completed his first cycle of concurrent chemoradiotherapy a month prior, with the initial tumor staging imaging studies showing no tumor invasion of the adjacent vascular structures.

**Figure 1:**
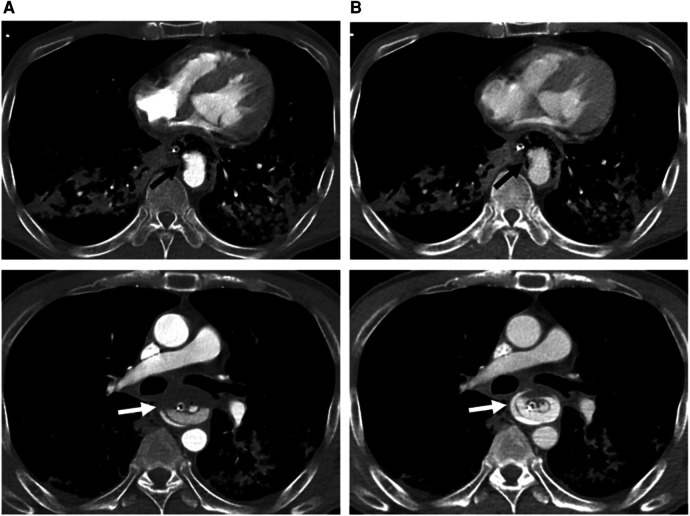
Axial computed tomography angiography of arterial (**A**) and venous (**B**) phases at two different levels below the carina, demonstrating a descending aortic pseudoaneurysm (black arrow) protruding into the esophagus (with a nasogastric tube *in situ*) through an aortoesophageal fistula (evidenced by direct communication of esophagus and aorta). There is a progressive increase in amount of contrast material (white arrow) in the esophagus, indicating rupture of the pseudoaneurysm and active bleeding.

**Figure 2:**
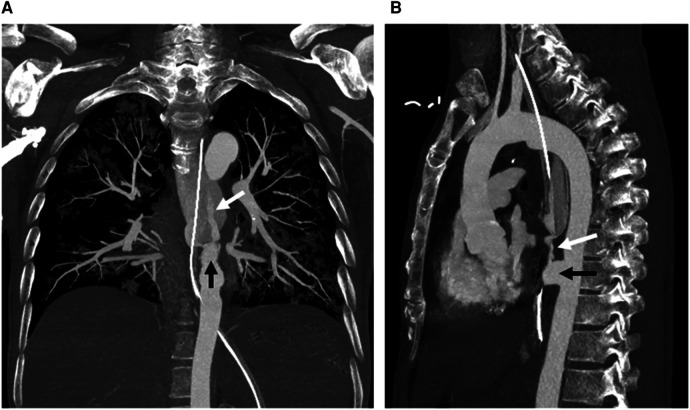
Coronal (**A**) and sagittal (**B**) maximum intensity projection images from computed tomography angiography show a descending aortic pseudoaneurysm (black arrow) at the level of the aortoesophageal fistula, complicated with rupture and contrast material extravasation (white arrow) into the esophagus (with a nasogastric tube *in situ*).

The patient was transfused with 6 units of packed cells in the ward. There was a symptom-free latent interval of 6 h, until the patient developed another bout of hematemesis and suffered a cardiovascular collapse while awaiting esophagoduodenoscopy. Cardiopulmonary resuscitation was performed in accordance with Advanced Cardiac Life Support protocols. He eventually achieved a return of spontaneous circulation after 36 min but required intubation and inotropic support. The patient was transfused with another 6 units of packed cells and 12 units of fresh frozen plasma. Computed tomography angiography (CTA) thereafter demonstrated an AEF with aortic pseudoaneurysm, as well as massive contrast extravasation at the distal esophagus suggestive of an active hemorrhage (Figures 1, 2). Yet another 6 units of packed cells, 6 units of fresh frozen plasma, and 12 units of platelets were transfused. Nevertheless, he finally succumbed to recurrent hematemesis leading to fatal exsanguination before definitive surgical intervention could be performed (9 h post herald bleed).

## Discussion

AEF is an extremely rare cardiovascular etiology of hematemesis and UGIB. As such, its recognition and diagnosis are challenging and may be delayed when such patients present to the ED. Without timely surgical treatment, AEF is almost always fatal; even with surgical intervention, AEF patients face a high mortality rate of 77% (5). Awareness of AEF as a possible diagnosis and consequently early identification of these patients presenting to the ED are therefore crucial in optimizing their clinical outcomes.

On retrospective review of our patient's clinical course, his progression illustrates the classical triad of an AEF (Chiari's triad)—midthoracic pain or dysphagia, a sentinel episode of minor hematemesis, and a symptom-free interval followed by fatal exsanguination due to recurrent hematemesis. The symptom-free interval during which there is spontaneous cessation of hematemesis has been described in up to 80% of AEF patients (6). In our case, this latent interval lasted approximately 6 h, possibly attributable to the transient occlusion of the AEF *via* a combination of periaortic hematomas, intravascular hypotension, and arterial wall spasm (7).

CTA is the investigation modality of choice to confirm the diagnosis of AEF, as it can objectively demonstrate contrast extravasation. OGDS may be useful in excluding other common causes of UGIB, as well as revealing a bulging pulsatile lesion or submucosal hematoma *via* direct visualization that is suggestive of bleeding into the esophageal wall (8, 9). Nevertheless, esophagoduodenoscopy can be hazardous due to the risk of dislodging the occluding periaortic hematoma responsible for hemostasis and precipitating fatal hemorrhage (10–12).

Clinicians should keep in mind that the diagnosis of AEF is possible in patients presenting with hematemesis to the ED, especially if they have underlying risk factors of previous aortic surgery, aortic aneurysms, and thoracic cancer. Patients suspected to have AEF can be prioritized for CTA to clinch the definitive diagnosis, and subsequent arrangements for surgical interventions can be expedited. While an earlier CTA may or may not have improved the survival chances of our patient, establishing the diagnosis quickly would have been beneficial in allowing our ED team to counsel the patient's family regarding his prognosis accordingly—in recognition of this, our ED now prioritizes CTA over esophagoduodenoscopy in patients who present with frank hematemesis and concurrently have known risk factors for AEF (aortic aneurysm, foreign body ingestion, and advanced esophageal carcinoma).

The definitive treatment of AEFs is usually a combination of aortic (thoracic endovascular aortic repair, graft replacement, graft repair) and esophageal (esophagectomy, esophageal stent, esophageal repair) surgeries (13). In the acute setting of massive hematemesis, the Sengstaken-Blakemore tube (SBT) has been reported to be effective in securing hemostasis *via* the gastroesophageal balloon's tamponade effect, to buy time for definitive surgery (7, 14). Nevertheless, deploying the SBT is not without its complications, such as aspiration pneumonitis, airway obstruction, mucosal ulceration, esophageal perforation, and broncho-esophageal fistulas (15–17).

Surgical treatment options of AEF include open surgery and thoracic endovascular aortic repair (TEVAR); the latter is a minimally invasive technique which deploys an endoluminal aortic stent to rapidly control the bleeding with a favorable 30-day mortality rate of 27.5% (18, 19). TEVAR however does not address the esophageal lesion in AEFs, which may form a nidus for infections and subsequently lead to stent graft infection, mediastinitis, sepsis, re-hemorrhage, and stroke (20, 21). In contrast, open surgery allows for the debridement of infected mediastinum and esophageal repair in addition to aortic wall reconstruction; the trade-off is a high operative mortality rate of up to 55% (22). Combining TEVAR as bridging therapy with follow-up definitive open repair has been found to yield the lowest mortality rate at 25% (19), though esophageal cancer patients like ours may benefit more from palliative esophageal stents with survival of up to 8 months (23).

## Conclusion

AEF is a rare and life-threatening cardiovascular cause of UGIB. It should be included in the list of differential diagnoses when evaluating patients presenting to the ED with hematemesis, especially if they have predisposing risk factors such as prior aortic or esophageal surgeries, aortic aneurysms, or thoracic malignancies. Patients suspected of having AEF should be prioritized for early CTA to expedite the diagnosis. Minimally invasive procedures such as SBT or TEVAR are pivotal to achieve initial hemostasis, which should be followed by definitive open surgery once the patient is stable.

## Data Availability

The original contributions presented in the study are included in the article/Supplementary Material, further inquiries can be directed to the corresponding author.
